# *N*-Cyclo­hexyl-4-meth­yl-*N*-propyl­benzene­sulfonamide

**DOI:** 10.1107/S1600536810006756

**Published:** 2010-03-03

**Authors:** Islam Ullah Khan, Zeeshan Haider, Muhammad Nadeem Arshad, Shahzad Sharif

**Affiliations:** a Materials Chemistry Laboratory, Department of Chemistry, GC University, Lahore, Pakistan

## Abstract

The title compound, C_16_H_25_NO_2_S, is a sulfonamide derivative with the substitution of propyl and cyclo­hexyl groups at the N atom. The least-squares plane through all six C atoms of the cyclo­hexyl ring forms a dihedral angle of 58.88 (12)° with the toluene ring. No hydrogen-bonding inter­actions are present in the crystal structure.

## Related literature

For the synthesis and related structures, see: Haider *et al.* (2009 ▶, 2010 ▶).
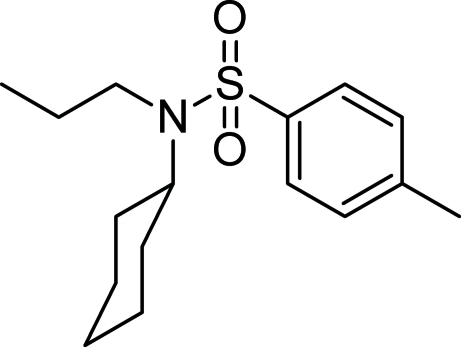


## Experimental

### 

#### Crystal data

C_16_H_25_NO_2_S
*M* *_r_* = 295.43
Monoclinic, 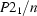

*a* = 7.8207 (5) Å
*b* = 25.2915 (16) Å
*c* = 8.3135 (6) Å
β = 102.411 (3)°
*V* = 1605.96 (19) Å^3^
*Z* = 4
Mo *K*α radiation
μ = 0.20 mm^−1^
*T* = 296 K
0.16 × 0.08 × 0.06 mm

#### Data collection

Bruker Kappa APEXII CCD diffractometer
Absorption correction: multi-scan (*SADABS*; Bruker, 2007 ▶) *T*_min_ = 0.980, *T*_max_ = 0.988
12946 measured reflections
3004 independent reflections
1271 reflections with *I* > 2σ(*I*)
*R*_int_ = 0.124

#### Refinement

*R*[*F* ^2^ > 2σ(*F* ^2^)] = 0.062
*wR*(*F* ^2^) = 0.128
*S* = 0.90
3003 reflections
183 parameters
H-atom parameters constrained
Δρ_max_ = 0.17 e Å^−3^
Δρ_min_ = −0.23 e Å^−3^

### 

Data collection: *APEX2* (Bruker, 2007 ▶); cell refinement: *SAINT* (Bruker, 2007 ▶); data reduction: *SAINT*; program(s) used to solve structure: *SHELXS97* (Sheldrick, 2008 ▶); program(s) used to refine structure: *SHELXL97* (Sheldrick, 2008 ▶); molecular graphics: *ORTEP-3 for Windows* (Farrugia, 1997 ▶) and *PLATON* (Spek, 2009 ▶); software used to prepare material for publication: *WinGX* (Farrugia, 1999 ▶) and *PLATON*.

## Supplementary Material

Crystal structure: contains datablocks I, global. DOI: 10.1107/S1600536810006756/om2321sup1.cif

Structure factors: contains datablocks I. DOI: 10.1107/S1600536810006756/om2321Isup2.hkl

Additional supplementary materials:  crystallographic information; 3D view; checkCIF report

## Figures and Tables

**Fig. 1.**
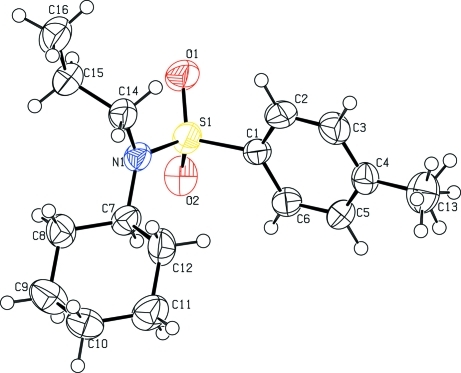
The labelled diagram of (I) with displacement ellipsoids drawn at the 50% probability level.

**Fig. 2.**
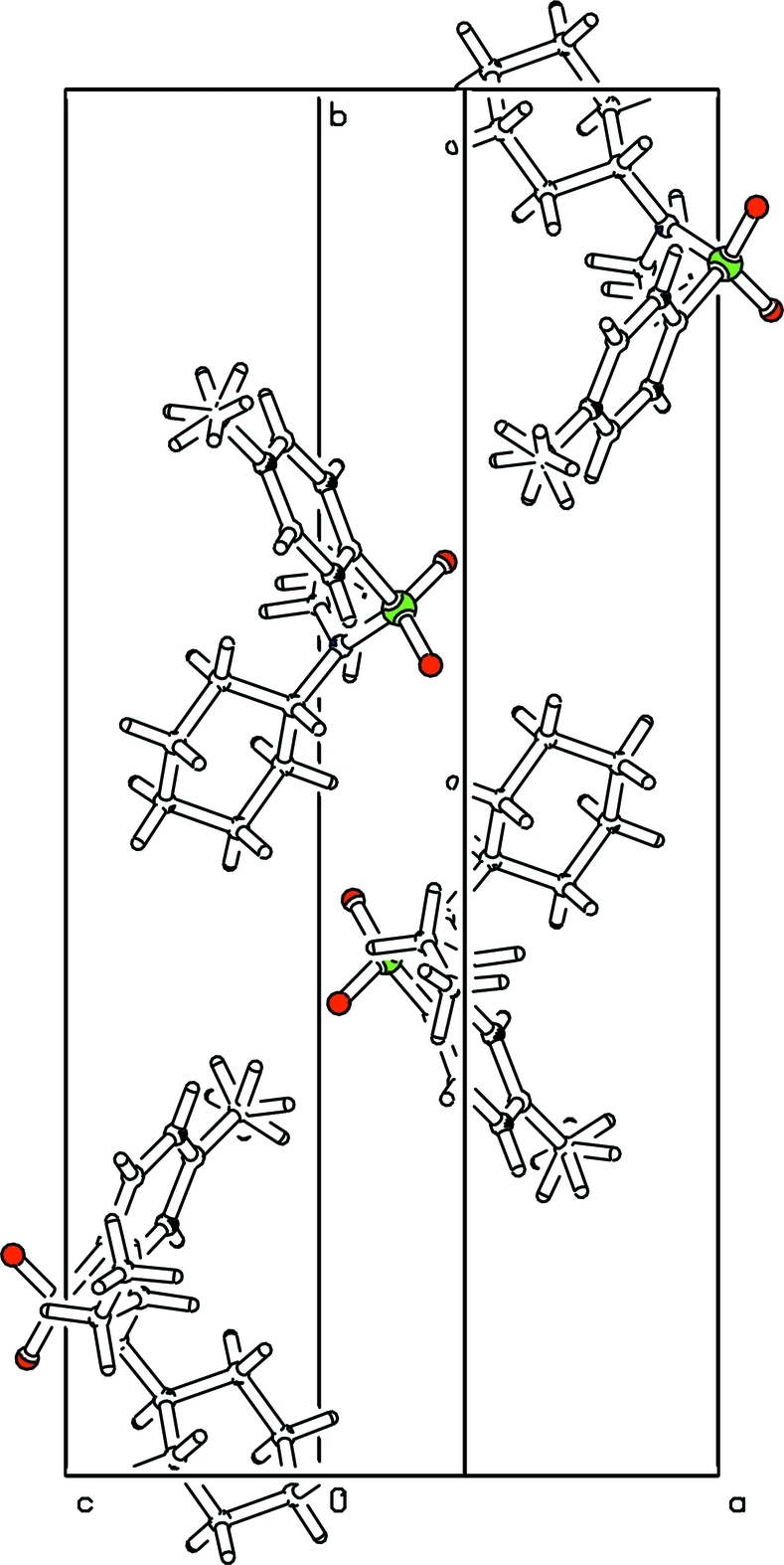
Unit cell packing for (I).

## supplementary crystallographic information

### Comment

The title compound (I) is an analogue to the structures (II) and (III) already published by our group (Haider *et al.*, 2009, 2010). The cyclohexyl ring adopts the chair form, and it is orientented with the least-squares plane of all six carbon atoms at the dihedral angle of 58.88 (12)° with respect to the aromatic ring. This angle compares well with the analogous angle in (III) (59.92 (6)°) but differs slightly from that found in (II) (50.13 (9)°). Likewise, in the title compound (see Fig. 2) and in (II), no hydrogen bonding interaction was observed in the structure.

### Experimental

A mixture of *N*-cyclohexyl-4-methyl benzene sulfonamide (1.089 g, 4.3 mmol), and sodium hydride (0.21 g, 8.6 mmol) in N, *N*-dimethylformamide (10 ml) was stirred at room temperature for half an hour followed by addition of propyl iodode ( 8.6 mmol). Stirring was continued further for a period of three hours and the contents were poured over crushed ice. The precipitated product was isolated, washed and crystallized from methanol solution by slow evaporation.

### Refinement

The C—H H-atoms were positioned gemetrically and were refined using a riding model with C–H = 0.93 Å and *U*_iso_(H) = 1.2 *U*_eq_ for aromatic (C), with C–H = 0.97 Å and *U*_iso_(H) = 1.2 *U*_eq_ for methylene (C), and with C–H = 0.98 Å and *U*_iso_(H) = 1.2 *U*_eq_ for (C7). The low angle reflection (0 2 0) was omitted in the final refinement and the H-atoms for methyl (C13) were refined at two positions using the HFIX 127 command in SHELXL97 with C–H = 0.96 Å and *U*_iso_(H) = 1.5 *U*_eq_ for (C13).

### Crystal data

**Table:**

C_16_H_25_NO_2_S	*F*(000) = 640
*M**_r_* = 295.43	*D*_x_ = 1.222 Mg m^−^^3^
Monoclinic, *P*2_1_/*n*	Mo *K*α radiation, λ = 0.71073 Å
Hall symbol: -P 2yn	Cell parameters from 856 reflections
*a* = 7.8207 (5) Å	θ = 2.6–17.1°
*b* = 25.2915 (16) Å	µ = 0.20 mm^−^^1^
*c* = 8.3135 (6) Å	*T* = 296 K
β = 102.411 (3)°	Needle, colorless
*V* = 1605.96 (19) Å^3^	0.16 × 0.08 × 0.06 mm
*Z* = 4	

### Data collection

**Table:**

Bruker Kappa APEXII CCD diffractometer	3004 independent reflections
Radiation source: fine-focus sealed tube	1271 reflections with *I* > 2σ(*I*)
graphite	*R*_int_ = 0.124
φ and ω scans	θ_max_ = 25.6°, θ_min_ = 1.6°
Absorption correction: multi-scan (*SADABS*; Bruker, 2007)	*h* = −9→9
*T*_min_ = 0.980, *T*_max_ = 0.988	*k* = −30→30
12946 measured reflections	*l* = −8→10

### Refinement

**Table:**

Refinement on *F*^2^	Primary atom site location: structure-invariant direct methods
Least-squares matrix: full	Secondary atom site location: difference Fourier map
*R*[*F*^2^ > 2σ(*F*^2^)] = 0.062	Hydrogen site location: inferred from neighbouring sites
*wR*(*F*^2^) = 0.128	H-atom parameters constrained
*S* = 0.90	*w* = 1/[σ^2^(*F*_o_^2^) + (0.0333*P*)^2^ + 0.8827*P*] where *P* = (*F*_o_^2^ + 2*F*_c_^2^)/3
3003 reflections	(Δ/σ)_max_ < 0.001
183 parameters	Δρ_max_ = 0.17 e Å^−^^3^
0 restraints	Δρ_min_ = −0.23 e Å^−^^3^

### Special details

**Table:**

Geometry. All s.u.'s (except the s.u. in the dihedral angle between two l.s. planes) are estimated using the full covariance matrix. The cell s.u.'s are taken into account individually in the estimation of s.u.'s in distances, angles and torsion angles; correlations between s.u.'s in cell parameters are only used when they are defined by crystal symmetry. An approximate (isotropic) treatment of cell s.u.'s is used for estimating s.u.'s involving l.s. planes.
Refinement. Refinement of *F*^2^ against ALL reflections. The weighted *R*-factor wR and goodness of fit *S* are based on *F*^2^, conventional *R*-factors *R* are based on *F*, with *F* set to zero for negative *F*^2^. The threshold expression of *F*^2^ > 2σ(*F*^2^) is used only for calculating *R*-factors(gt) etc. and is not relevant to the choice of reflections for refinement. *R*-factors based on *F*^2^ are statistically about twice as large as those based on *F*, and *R*- factors based on ALL data will be even larger.

### Fractional atomic coordinates and isotropic or equivalent isotropic displacement parameters (Å^2^)

**Table:**

	*x*	*y*	*z*	*U*_iso_*/*U*_eq_	Occ. (<1)
S1	−0.12769 (14)	0.12574 (4)	0.82665 (13)	0.0507 (3)	
O1	−0.1615 (4)	0.15917 (11)	0.9551 (3)	0.0687 (9)	
O2	−0.2485 (3)	0.08478 (11)	0.7610 (3)	0.0626 (8)	
N1	0.0618 (4)	0.09828 (12)	0.8964 (4)	0.0444 (8)	
C1	−0.1095 (5)	0.16743 (15)	0.6605 (5)	0.0410 (10)	
C2	−0.0304 (5)	0.21596 (16)	0.6879 (5)	0.0570 (12)	
H2	0.0079	0.2283	0.7950	0.068*	
C3	−0.0076 (5)	0.24655 (16)	0.5559 (6)	0.0583 (12)	
H3	0.0477	0.2792	0.5761	0.070*	
C4	−0.0647 (5)	0.22991 (16)	0.3951 (5)	0.0475 (11)	
C5	−0.1461 (5)	0.18147 (16)	0.3710 (5)	0.0510 (11)	
H5	−0.1880	0.1695	0.2639	0.061*	
C6	−0.1676 (5)	0.14986 (15)	0.5021 (5)	0.0464 (11)	
H6	−0.2211	0.1170	0.4824	0.056*	
C7	0.1201 (5)	0.05534 (15)	0.7993 (5)	0.0479 (11)	
H7	0.0146	0.0388	0.7337	0.058*	
C8	0.2179 (5)	0.01308 (14)	0.9125 (5)	0.0559 (12)	
H8A	0.1448	0.0002	0.9847	0.067*	
H8B	0.3230	0.0282	0.9804	0.067*	
C9	0.2676 (6)	−0.03285 (16)	0.8126 (6)	0.0709 (14)	
H9A	0.3344	−0.0587	0.8867	0.085*	
H9B	0.1620	−0.0500	0.7525	0.085*	
C10	0.3749 (6)	−0.01397 (16)	0.6925 (5)	0.0611 (13)	
H10A	0.3995	−0.0436	0.6269	0.073*	
H10B	0.4857	0.0000	0.7531	0.073*	
C11	0.2786 (6)	0.02856 (18)	0.5800 (5)	0.0739 (14)	
H11A	0.1737	0.0137	0.5110	0.089*	
H11B	0.3526	0.0416	0.5089	0.089*	
C12	0.2289 (6)	0.07402 (16)	0.6810 (5)	0.0654 (13)	
H12A	0.3346	0.0908	0.7423	0.078*	
H12B	0.1635	0.1002	0.6073	0.078*	
C13	−0.0380 (6)	0.26366 (17)	0.2532 (5)	0.0729 (14)	
H13A	0.0274	0.2947	0.2949	0.109*	0.50
H13B	−0.1497	0.2739	0.1878	0.109*	0.50
H13C	0.0254	0.2439	0.1865	0.109*	0.50
H13D	−0.0920	0.2470	0.1512	0.109*	0.50
H13E	0.0851	0.2678	0.2583	0.109*	0.50
H13F	−0.0900	0.2978	0.2597	0.109*	0.50
C14	0.1931 (5)	0.12873 (15)	1.0130 (4)	0.0508 (11)	
H14A	0.1650	0.1660	0.9987	0.061*	
H14B	0.3067	0.1233	0.9866	0.061*	
C15	0.2050 (5)	0.11426 (15)	1.1910 (5)	0.0560 (12)	
H15A	0.2365	0.0772	1.2070	0.067*	
H15B	0.0913	0.1191	1.2179	0.067*	
C16	0.3390 (6)	0.14751 (16)	1.3061 (5)	0.0729 (14)	
H16A	0.4510	0.1438	1.2774	0.109*	
H16B	0.3473	0.1359	1.4174	0.109*	
H16C	0.3038	0.1839	1.2965	0.109*	

### Atomic displacement parameters (Å^2^)

**Table:**

	*U*^11^	*U*^22^	*U*^33^	*U*^12^	*U*^13^	*U*^23^
S1	0.0461 (7)	0.0630 (7)	0.0445 (7)	0.0111 (7)	0.0127 (5)	0.0063 (6)
O1	0.076 (2)	0.086 (2)	0.050 (2)	0.0307 (17)	0.0248 (16)	−0.0003 (16)
O2	0.0441 (18)	0.072 (2)	0.071 (2)	−0.0052 (16)	0.0099 (15)	0.0123 (16)
N1	0.042 (2)	0.053 (2)	0.036 (2)	0.0063 (17)	0.0035 (16)	0.0006 (16)
C1	0.041 (3)	0.044 (2)	0.038 (3)	0.005 (2)	0.006 (2)	−0.004 (2)
C2	0.067 (3)	0.054 (3)	0.044 (3)	0.002 (2)	−0.002 (2)	−0.013 (2)
C3	0.066 (3)	0.042 (3)	0.061 (3)	−0.007 (2)	0.002 (3)	−0.002 (2)
C4	0.042 (3)	0.051 (3)	0.048 (3)	0.006 (2)	0.007 (2)	0.007 (2)
C5	0.059 (3)	0.054 (3)	0.036 (3)	0.003 (2)	0.002 (2)	−0.001 (2)
C6	0.047 (3)	0.044 (2)	0.045 (3)	−0.005 (2)	0.004 (2)	−0.004 (2)
C7	0.044 (3)	0.060 (3)	0.041 (3)	0.004 (2)	0.010 (2)	0.000 (2)
C8	0.063 (3)	0.053 (3)	0.058 (3)	0.004 (2)	0.027 (2)	0.013 (2)
C9	0.073 (4)	0.052 (3)	0.097 (4)	0.005 (3)	0.040 (3)	0.000 (3)
C10	0.056 (3)	0.058 (3)	0.073 (3)	0.002 (2)	0.024 (3)	−0.009 (2)
C11	0.070 (4)	0.100 (4)	0.057 (3)	0.019 (3)	0.026 (3)	0.002 (3)
C12	0.072 (3)	0.073 (3)	0.058 (3)	0.026 (3)	0.028 (3)	0.023 (2)
C13	0.074 (3)	0.075 (3)	0.070 (3)	−0.007 (3)	0.016 (3)	0.016 (3)
C14	0.052 (3)	0.054 (3)	0.046 (3)	0.006 (2)	0.007 (2)	0.004 (2)
C15	0.060 (3)	0.064 (3)	0.043 (3)	−0.003 (2)	0.008 (2)	0.002 (2)
C16	0.089 (4)	0.075 (3)	0.048 (3)	−0.011 (3)	−0.001 (3)	0.004 (2)

### Geometric parameters (Å, °)

**Table:**

S1—O2	1.429 (3)	C9—H9B	0.9700
S1—O1	1.430 (3)	C10—C11	1.516 (5)
S1—N1	1.627 (3)	C10—H10A	0.9700
S1—C1	1.767 (4)	C10—H10B	0.9700
N1—C14	1.469 (4)	C11—C12	1.522 (5)
N1—C7	1.482 (4)	C11—H11A	0.9700
C1—C2	1.371 (5)	C11—H11B	0.9700
C1—C6	1.372 (5)	C12—H12A	0.9700
C2—C3	1.385 (5)	C12—H12B	0.9700
C2—H2	0.9300	C13—H13A	0.9600
C3—C4	1.381 (5)	C13—H13B	0.9600
C3—H3	0.9300	C13—H13C	0.9600
C4—C5	1.375 (5)	C13—H13D	0.9600
C4—C13	1.507 (5)	C13—H13E	0.9600
C5—C6	1.391 (5)	C13—H13F	0.9600
C5—H5	0.9300	C14—C15	1.507 (5)
C6—H6	0.9300	C14—H14A	0.9700
C7—C12	1.508 (5)	C14—H14B	0.9700
C7—C8	1.518 (5)	C15—C16	1.513 (5)
C7—H7	0.9800	C15—H15A	0.9700
C8—C9	1.526 (5)	C15—H15B	0.9700
C8—H8A	0.9700	C16—H16A	0.9600
C8—H8B	0.9700	C16—H16B	0.9600
C9—C10	1.513 (5)	C16—H16C	0.9600
C9—H9A	0.9700		
			
O2—S1—O1	120.02 (18)	C10—C11—C12	110.3 (4)
O2—S1—N1	107.69 (17)	C10—C11—H11A	109.6
O1—S1—N1	106.65 (17)	C12—C11—H11A	109.6
O2—S1—C1	106.95 (18)	C10—C11—H11B	109.6
O1—S1—C1	106.83 (18)	C12—C11—H11B	109.6
N1—S1—C1	108.25 (16)	H11A—C11—H11B	108.1
C14—N1—C7	119.4 (3)	C7—C12—C11	111.7 (4)
C14—N1—S1	117.7 (2)	C7—C12—H12A	109.3
C7—N1—S1	118.8 (3)	C11—C12—H12A	109.3
C2—C1—C6	119.6 (4)	C7—C12—H12B	109.3
C2—C1—S1	120.9 (3)	C11—C12—H12B	109.3
C6—C1—S1	119.4 (3)	H12A—C12—H12B	107.9
C1—C2—C3	119.9 (4)	C4—C13—H13A	109.5
C1—C2—H2	120.0	C4—C13—H13B	109.5
C3—C2—H2	120.0	H13A—C13—H13B	109.5
C4—C3—C2	121.8 (4)	C4—C13—H13C	109.5
C4—C3—H3	119.1	H13A—C13—H13C	109.5
C2—C3—H3	119.1	H13B—C13—H13C	109.5
C5—C4—C3	117.1 (4)	C4—C13—H13D	109.5
C5—C4—C13	121.9 (4)	H13A—C13—H13D	141.1
C3—C4—C13	121.0 (4)	H13B—C13—H13D	56.3
C4—C5—C6	121.8 (4)	H13C—C13—H13D	56.3
C4—C5—H5	119.1	C4—C13—H13E	109.5
C6—C5—H5	119.1	H13A—C13—H13E	56.3
C1—C6—C5	119.7 (4)	H13B—C13—H13E	141.1
C1—C6—H6	120.1	H13C—C13—H13E	56.3
C5—C6—H6	120.1	H13D—C13—H13E	109.5
N1—C7—C12	114.1 (3)	C4—C13—H13F	109.5
N1—C7—C8	110.6 (3)	H13A—C13—H13F	56.3
C12—C7—C8	110.2 (3)	H13B—C13—H13F	56.3
N1—C7—H7	107.2	H13C—C13—H13F	141.1
C12—C7—H7	107.2	H13D—C13—H13F	109.5
C8—C7—H7	107.2	H13E—C13—H13F	109.5
C7—C8—C9	110.6 (3)	N1—C14—C15	114.1 (3)
C7—C8—H8A	109.5	N1—C14—H14A	108.7
C9—C8—H8A	109.5	C15—C14—H14A	108.7
C7—C8—H8B	109.5	N1—C14—H14B	108.7
C9—C8—H8B	109.5	C15—C14—H14B	108.7
H8A—C8—H8B	108.1	H14A—C14—H14B	107.6
C10—C9—C8	111.2 (3)	C14—C15—C16	112.0 (3)
C10—C9—H9A	109.4	C14—C15—H15A	109.2
C8—C9—H9A	109.4	C16—C15—H15A	109.2
C10—C9—H9B	109.4	C14—C15—H15B	109.2
C8—C9—H9B	109.4	C16—C15—H15B	109.2
H9A—C9—H9B	108.0	H15A—C15—H15B	107.9
C9—C10—C11	111.0 (3)	C15—C16—H16A	109.5
C9—C10—H10A	109.4	C15—C16—H16B	109.5
C11—C10—H10A	109.4	H16A—C16—H16B	109.5
C9—C10—H10B	109.4	C15—C16—H16C	109.5
C11—C10—H10B	109.4	H16A—C16—H16C	109.5
H10A—C10—H10B	108.0	H16B—C16—H16C	109.5
			
O2—S1—N1—C14	−161.9 (3)	C2—C1—C6—C5	−0.2 (6)
O1—S1—N1—C14	−31.8 (3)	S1—C1—C6—C5	−177.0 (3)
C1—S1—N1—C14	82.8 (3)	C4—C5—C6—C1	1.2 (6)
O2—S1—N1—C7	41.0 (3)	C14—N1—C7—C12	−64.2 (5)
O1—S1—N1—C7	171.1 (3)	S1—N1—C7—C12	92.5 (4)
C1—S1—N1—C7	−74.3 (3)	C14—N1—C7—C8	60.7 (4)
O2—S1—C1—C2	167.1 (3)	S1—N1—C7—C8	−142.6 (3)
O1—S1—C1—C2	37.4 (4)	N1—C7—C8—C9	176.3 (3)
N1—S1—C1—C2	−77.1 (3)	C12—C7—C8—C9	−56.6 (5)
O2—S1—C1—C6	−16.1 (3)	C7—C8—C9—C10	56.6 (5)
O1—S1—C1—C6	−145.8 (3)	C8—C9—C10—C11	−56.2 (5)
N1—S1—C1—C6	99.7 (3)	C9—C10—C11—C12	55.7 (5)
C6—C1—C2—C3	−0.9 (6)	N1—C7—C12—C11	−177.7 (3)
S1—C1—C2—C3	175.9 (3)	C8—C7—C12—C11	57.2 (5)
C1—C2—C3—C4	0.9 (6)	C10—C11—C12—C7	−56.7 (5)
C2—C3—C4—C5	0.1 (6)	C7—N1—C14—C15	−104.2 (4)
C2—C3—C4—C13	−179.7 (4)	S1—N1—C14—C15	98.8 (3)
C3—C4—C5—C6	−1.2 (6)	N1—C14—C15—C16	−178.8 (3)
C13—C4—C5—C6	178.7 (4)		
